# A rare case of circulating anaplastic lymphoma kinase-negative anaplastic large cell lymphoma

**DOI:** 10.1016/j.htct.2025.103743

**Published:** 2025-02-15

**Authors:** Radu Chiriac, Lucile Baseggio

**Affiliations:** Laboratoire d'hématologie biologique, Hospices Civils de Lyon, Pierre-Bénite, France

A 71-year-old female presented with B symptoms, splenomegaly, and bowel obstruction. Her blood count showed leukocytosis (79 × 10^9^/L), anemia (100 g/L), and thrombocytopenia (70 × 10^9^/L).

A blood smear revealed large lymphomatous cells with distinctive nuclei and cytoplasmic granules. Flow cytometry identified large CD4^+^ *T*-cells with abnormal CD3 and CD7 loss, a cytotoxic profile, and dimCD30 expression. A lymph node biopsy confirmed anaplastic large cell lymphoma (ALCL), anaplastic lymphoma kinase (ALK)-negative, characterized by strong CD30 expression.

Peripheral blood involvement in ALK-negative ALCL is rare, highlighting diagnostic challenges and the need for accurate identification to ensure proper treatment (Figure 1).^1^^,^^2^

**Figure 1:**
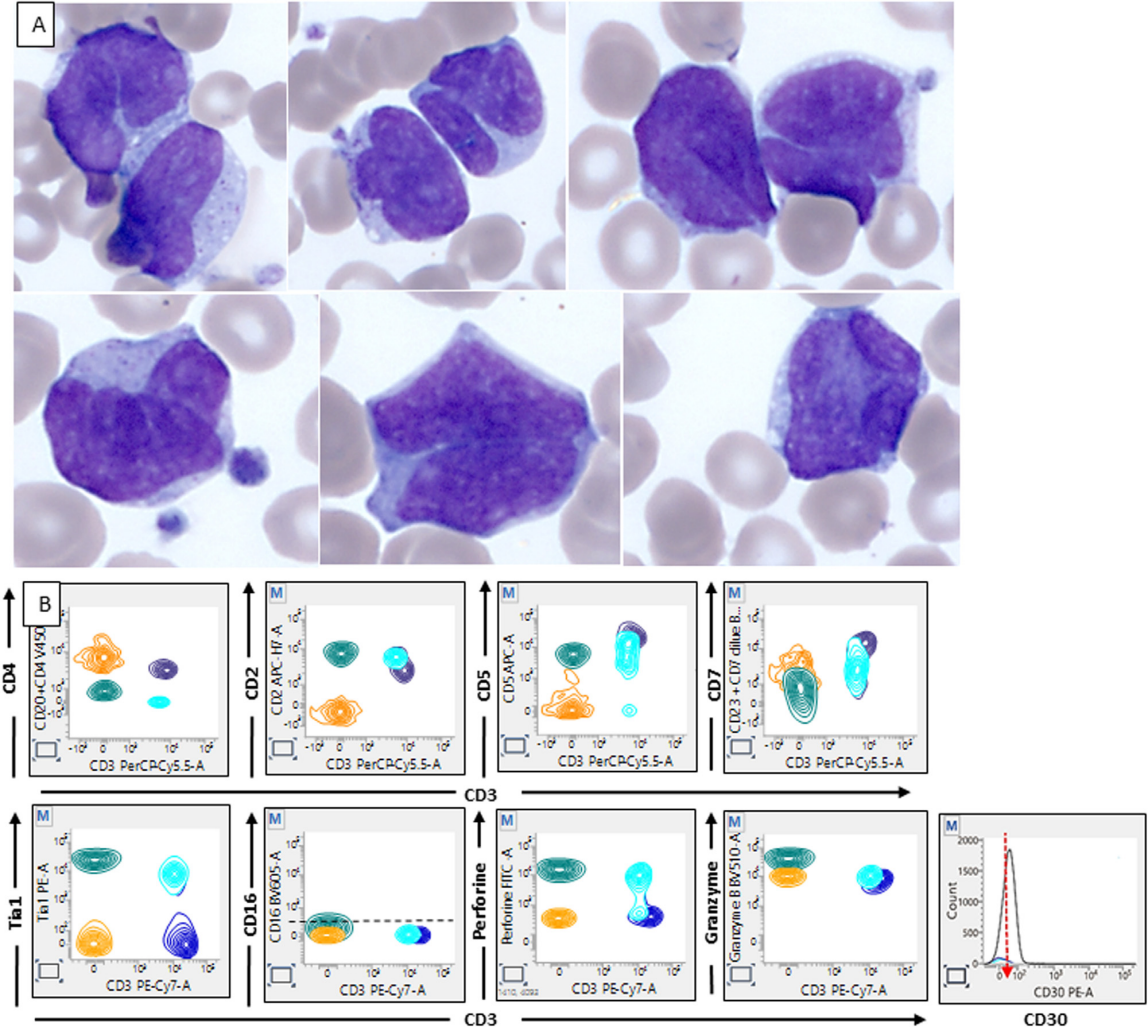
Panel A: May-Grunwald Giemsa (x100 magnification): blood smear reveals large lymphomatous cells with highly contoured, occasionally notched, and sometimes "flower-like" nuclei; Panel B: green plot: flow cytometric profile, CD4^+^*T*-cells with abnormal loss of CD3, CD7 expression, and a cytotoxic profile.

## Author contributions

Lucile Baseggio and Radu Chiriac wrote the manuscript and conducted the cytological and flow cytometric studies. Both authors contributed to the final manuscript.

## Data availability

Data sharing is not applicable to this article as no new data were created or analyzed in this study.

## Ethics approval statement

This manuscript respects the ethic policy of CHU Lyon for the treatment of human research participants.

## Patient consent statement

No patient-identifying data were used. The authors did not obtain written informed consent from the patient but the patient did not object to his data being used for research purposes (as required by ethic policy of CHU Lyon).

## Permission to reproduce material from other sources

The authors declare no use of third-party material in this study for which formal permission is required.

## Clinical trial registration

Not applicable.

## Conflicts of interest

The authors have no conflict of interest.
